# Passive and Wireless All‐Textile Wearable Sensor System

**DOI:** 10.1002/advs.202206665

**Published:** 2023-05-19

**Authors:** Valeria Galli, Sunil Kumar Sailapu, Tyler J. Cuthbert, Chakaveh Ahmadizadeh, Brett C. Hannigan, Carlo Menon

**Affiliations:** ^1^ Biomedical and Mobile Health Technology (BMHT) Group Department of Health Sciences and Technology ETH Zürich Lengghalde 5 Zürich 8008 Switzerland

**Keywords:** battery‐free, digital health, passive, smart textile, wireless sensors

## Abstract

Mobile health technology and activity tracking with wearable sensors enable continuous unobtrusive monitoring of movement and biophysical parameters. Advancements in clothing‐based wearable devices have employed textiles as transmission lines, communication hubs, and various sensing modalities; this area of research is moving towards complete integration of circuitry into textile components. A current limitation for motion tracking is the need for communication protocols demanding physical connection of textile with rigid devices, or vector network analyzers (VNA) with limited portability and lower sampling rates. Inductor–capacitor (LC) circuits are ideal candidates as textile sensors can be easily implemented with textile components and allow wireless communication. In this paper, the authors report a smart garment that can sense movement and wirelessly transmit data in real time. The garment features a passive LC sensor circuit constructed of electrified textile elements that sense strain and communicate through inductive coupling. A portable, lightweight reader (fReader) is developed for achieving a faster sampling rate than a downsized VNA to track body movement, and for wirelessly reading sensor information suitable for deployment with a smartphone. The smart garment–fReader system monitors human movement in real‐time and exemplifies the potential of textile‐based electronics moving forward.

## Graphical abstract

A movement‐tracking wearable device is presented that employs all‐textile components for sensing and wireless communication with an external custom reader. An LC circuit is fabricated with a sensing textile capacitor, stitched transmission lines, and an embroidered inductive coil. The custom reader overcomes the limitations of vector network analyzers, with the capability of tracking movement in real time.

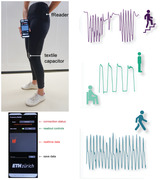

## 1 Introduction

Mobile health (mHealth) opens the possibility for personalized, remote healthcare monitoring, and risk assessment.^[^ ^1^, ^2^ ^]^ The increasing number of miniaturized mHealth technologies^[^ ^3^ ^]^ in the form of portable,^[^ ^4^ ^]^ implantable,^[^ ^5^, ^6^ ^]^ and wearable devices demonstrate the ability to sense, capture, process, and transmit various physiological parameters to improve wellness. The key value associated with mHealth technologies relies on the feasibility of acquiring relevant physiological data in a naturalistic, unconstrained, and ideally unperceived manner. Potential mHealth solutions that primarily focus on biomechanical movement should acquire and transmit information without restricting human motion and be comfortable to use. Tracking human motion with wearables has gained considerable attention because of its significance in the fields of rehabilitation and physiotherapy, prosthesis control, gait, and long‐term physiological signal (e.g., calorie expenditure, heart, and respiration rate) analysis.^[^ ^7^, ^8^ ^]^ Specifically, monitoring joint angles provides valuable clinical data through non‐invasive measurements. For instance, the knee bending angle has been largely associated with the range of motion and overall functionality of the joint.^[^ ^9^ ^]^ Clothing‐based solutions present an opportunity to access information related to different degrees of movement in an unobtrusive manner. However, a seamless integration of sensing elements along with a convenient readout approach remains unavailable, yet crucial for adoption.

Wearables can be broadly divided into three categories: accessories like smartwatches, wristbands, earphones, and glasses;^[^ ^10^ ^]^ devices in direct contact with the body like epidermal devices,^[^ ^2^ ^]^ (skin patches,^[^ ^11^ ^]^ electronic skin^[^ ^12^ ^]^), contact lenses,^[^ ^13^ ^]^ tooth patches;^[^ ^14^ ^]^ and devices integrated into garments. The advantages of employing textile‐based wearable devices (i.e., smart clothing) are their natural contact and their ability to form to the body. Textile‐based sensing platforms can sense at many different locations simultaneously^[^ ^15^ ^]^ as opposed to implantable devices—and are the least intrusive when compared to accessories or on‐skin devices. These features make textile‐based wearables more likely to be accepted for everyday use than any other smart health monitoring device.^[^ ^16^ ^]^ Particularly, eliminating bulky power and electronics modules, rigid components, and wired connections will reduce weight, eliminate problematic connections, and increase ease of daily use. Recent efforts integrating wireless sensing platforms in textiles majorly include two categories: i) employing common wireless communication protocols with commercially available sensor tags or ii) custom‐made sensing platforms with inductance‐capacitance (LC) circuitry that relies on resonance frequency shifts for signal detection. In the first category, the sensor tags are usually equipped with a dedicated chip to enable Bluetooth,^[^ ^17^, ^18^, ^19^ ^]^ near‐field communication (NFC),^[^ ^20^, ^21^, ^22^, ^23^, ^24^ ^]^ or radio frequency identification (RFID)^[^ ^25^ ^]^ communication. Here, although functionalized garments have served to interconnect multiple sensor tags at different locations on a garment,^[^ ^19^, ^22^, ^24^ ^]^ these examples employ rigid components in their design. The sensors further require establishing a direct physical connection (wire‐based) with microcontrollers or wireless modules and power sources to measure, process, and transmit information.^[^ ^26^, ^27^ ^]^ However, the ultimate step for smart clothing is realizing all sensors, electrical components/connections, and communication in a full textile‐form as part of the garment itself. Recent attempts to use textile RFID^[^ ^28^ ^]^ or NFC antennas^[^ ^29^ ^]^ move in the direction of all‐textile sensor tags, they are yet to explore motion tracking. Furthermore, having a complete textile system removes the need for external electrical connections and eases the burden of washing the garments as there are no soldered/mounted electronic components to secure or remove.

The second category of wireless sensing platforms in textiles typically employs inductive coupling to read out signals passively (i.e., no battery power) with LC resonators as sensors,^[^ ^30^ ^]^ thus avoiding the need for a wired connection with rigid silicon‐based components. The LC circuit‐based sensors require only two components—inductor (L) and capacitor (C), making it easily implementable in a fully‐textile form. The modulation in the circuit's sensing component (L or C) induced by the measured parameter (e.g., motion) causes a resonance frequency shift of the LC sensor circuit, which can be wirelessly read through inductive coupling. Although studies in the past employed LC sensor circuits in wearables,^[^ ^31^, ^32^, ^33^ ^]^ the use of full‐textile sensors with wireless communication capabilities remains scarce. Few studies reported the fabrication of stimuli‐responsive textile‐based capacitors to induce a resonance frequency shift in the LC circuit when exposed to humidity or pressure.^[^ ^34^, ^35^ ^]^ With the emergence of so‐called e‐textiles^[^ ^36^ ^]^ or textronics,^[^ ^16^ ^]^ novel materials and techniques have aided in conferring conductivity to standard fabrics at the level of single fiber, yarn (filament), or fabric.^[^ ^37^ ^]^ Electronic circuitry elements such as capacitors and inductors can be fabricated, and seamlessly integrated into garments using relatively accessible conventional techniques such as sewing or embroidery. Employing materials and textile patterns with inherent flexibility and stretchability may further ensure the desired robustness for tracking medium to fast‐paced events such as walking, running, or climbing stairs. For wireless sensing with passive LC sensors, the external reader must be in close proximity to the inductor of the LC sensor. The resonance frequency shifts with these LC sensors have been previously recorded with a coupled inductor connected to benchtop vector network analyzers (VNA),^[^ ^5^, ^31^, ^34^, ^35^, ^38^ ^]^ or impedance analyzers.^[^ ^39^ ^]^ These readers are not suitable for wearable applications due to their bulky nature (even downsized VNAs) and cannot be integrated into daily living.^[^ ^25^ ^]^ Moreover, the sampling rate for downsized VNAs is low and requires a tradeoff between resolution in time and the scanned frequency range. These limitations in portability and sampling rate call for alternative wireless readout methodologies, especially suitable for tracking moderate to fast‐paced movements with textiles. Other wireless readout techniques with textile‐based capacitive sensors towards continuous motion tracking still require a direct physical connection between the sensor and rigid components like capacitance‐to‐digital converters, microcontrollers, and Bluetooth modules.^[^ ^35^ ^]^ Thereby, mHealth technology for activity‐tracking with a garment requires an LC sensor in a fully‐textile template to be reliably read with a lightweight compatible wireless reader.

**Figure 1:**
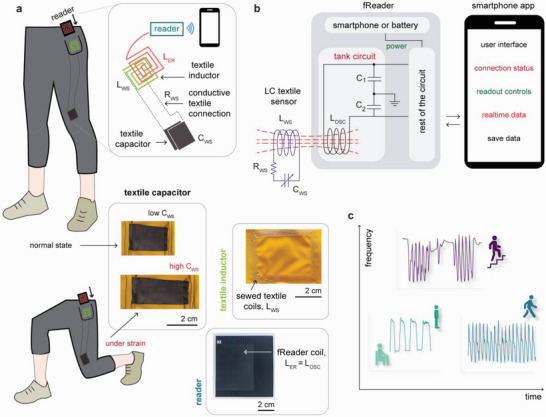
Passive‐wireless sensing system for motion‐tracking with smart garment. a) The scheme illustrates the smart garment with a parallel‐plate textile capacitive sensor (C_WS_) mounted above the kneecap and connected to a textile inductor (L_WS_) via conductive thread. The textile inductor near the vicinity of the garment pocket inductively couples with an external reader (L_ER_) placed in the pocket. The reader measures the frequency response from the strain‐induced capacitance changes in the textile capacitor via coupling with the textile inductor. b) Scheme depicting the readout process with a custom‐built reader (fReader) that communicates the frequency response related to strain to the smartphone. c) Representative output signals for different activities using the smart garment recorded wirelessly with the fReader.

In this work, we report a wireless sensing wearable system with a smart garment featuring an LC sensor circuit—fabricated exclusively with textile elements—and a custom‐built reader to record the sensor response via inductive coupling (**Figure** **1**). The passive LC textile sensor is composed of an inductor (*L_WS_*) sewn with conductive thread and a parallel‐plate capacitor (*C_WS_*) fabricated with stretchable conductive textiles, interconnected through a conductive haberdashery. The LC textile sensor responds to strain through capacitance, changing the resonance frequency. We built a lightweight, low‐cost Colpitts oscillator‐based frequency reader (fReader) to wirelessly read out the strain‐induced capacitance changes in a convenient way by placing it comfortably in a garment's pocket (Figure 1a). Unlike a VNA that relies on a frequency sweep, the circuit strategy employed in fReader is simple with detection based on a shift in the oscillator frequency (Figure 1b). It further provides information in real‐time by wirelessly communicating with a smartphone through a custom‐built application. As a proof of concept, we employed the smart garment to track various body movements by passively measuring the strain‐induced capacitance changes (Figure 1c). The sensing approach with fReader delivered a faster sampling rate to track human motion with the fabricated textile LC sensor when compared to portable yet bulky VNAs. Advancing such sensing modalities would expand the possible tools for applied researchers looking to employ wearable devices in physical rehabilitation, athletic performance improvement, injury prevention, and well‐being.

## 2 Results and Discussion

### 2.1 Design and Sensing Strategy of All‐Textile Sensor System

Physiological movements involve medium to fast‐paced displacements of body parts. Textile‐based motion‐tracking wearable systems intended for daily use must be comfortable, unobtrusive, and robust to identify these movements. Strain is a convenient parameter to track body motion through smart clothing as it aligns with the natural deformation of tight‐fitting clothes during movement.

To build a fully textile LC circuit sensitive to strain, modulation of the resonance frequency is required in response to a change in the geometry of either the inductor or the capacitor. In our case, we chose to rely on the change in capacitance rather than inductance for two main reasons. First, since the textile inductor is crucial for coupling with an external reader coil, eliminating any change in its inductance when strained would maintain a constant coupling intensity and facilitate good wireless signal quality. Second, it has been previously observed that inductive strain sensors have limited sensitivity to strain when compared to capacitive or resistive ones.^[^ ^40^ ^]^ Hence, we employed parallel plate capacitive strain sensors that rely on geometrical changes to modulate capacitance and are advantageous for constructing textile sensors since conductive textiles can be made using a myriad of methods. Our textile LC sensor featured a bare minimum of elements on the garments—a strain‐sensitive textile capacitor electrically connected to a fixed‐value textile inductor (Figure 1a). The resistance of the LC sensor is simply the equivalent series resistance of the textile inductor, capacitor, and the conductive thread connecting them.

The strain‐induced changes in the capacitance of the textile capacitor can be obtained by measuring the resonance frequency (*f_res_*) of the LC circuit, defined as

(1) $$f_{\mathrm{res}}=\frac{1}{2\pi \sqrt{L_{\mathrm{WS}}{\;C}_{\mathrm{WS}}}}$$

where *L_WS_* and *C_WS_* correspond to the inductance and capacitance of the textile inductor and capacitor, respectively. Here, the reader can typically be a VNA linked to an external coil that couples with the textile inductor to extract this resonance‐related information. However, downsized/portable VNAs are not suitable as a communication device for motion‐tracking with textile‐based wearables as they are bulky to carry around and are slow to track movement. So, we looked to eliminate the main drawbacks of the VNA by designing a lightweight, affordable frequency reader (fReader, Figure 1b) by adopting a different readout principle. In contrast to a VNA that constantly sweeps frequencies to identify *f_res_*, the fReader contains a Colpitts oscillator that rapidly modulates its output oscillating frequency (*f_osc_*) and reports a specific value based on the strain‐induced capacitance changes of the textile capacitor. Considering an ideal case of an air‐core transformer model, when the oscillator coil (*L_osc_* = *L_ER_*) couples with a textile inductor, the frequency of a Colpitts oscillator can be expressed as

(2) $$f_{\mathrm{osc}}=\frac{1}{2\pi \sqrt{L_{\mathrm{ER}}C_{L}}};C_{L}=C_{\mathrm{osc}}+n^{2}C_{\mathrm{ws}}$$

where *C_osc_* is the capacitance of the tank circuit in the oscillator and *n* is the ratio of windings in the transformer model. The fReader can establish communication wirelessly through a custom‐designed software application with devices like smartphones and can work either independently on battery or by drawing power from the smartphone itself.

### 2.2 Fabrication of the LC Textile Sensor

The textile capacitor was designed to sit above the kneecap, where maximum strain occurred upon joint flexion/extension, without it being compressed when flexing the knee.^[^ ^41^ ^]^ The textile inductor was designed with suitable geometry to fit a garment's pocket for effective coupling with the external reader's inductor for a stable response. To manufacture the textile capacitor and inductor in a straightforward manner with a traditional sewing machine, we sought suitable materials with high conductivity, mechanical strength, and natural textile feeling. Two electrodes made of conductive spandex sandwiched two layers of non‐conductive base spandex resulting in a parallel‐plate capacitor configuration (Figure 1a, *C_WS_*). The similar mechanical properties of the base spandex and conductive spandex allowed for preserving both textiles’ stretchability. We fabricated the textile inductor by sewing a highly conductive thread in planar rectangular loops with equal spacing between each turn of the loop (Figure 1a, *L_WS_*). The inductor coil and stretchable capacitor were connected electrically with conductive thread.

Even by utilizing highly conductive materials, typical capacitances and inductances obtained with textile‐based wearables in the past are in the order of tens of picoFarads^[^ ^42^, ^43^ ^]^ and a few microHenrys.^[^ ^44^, ^45^ ^]^ To assess the basic nature of the fabricated elements and to predict the resonance frequency characteristics of our LC textile sensor, we independently assessed the impedance response of the textile capacitor and inductor under no strain. The textile capacitor and inductor displayed a typical capacitance and inductance of around 15.8 pF and 2.3 µH, respectively (Figures S1 and S2, Supporting Information). The theoretical resonance frequency (*f_res_*) with the combined textile LC sensor under no strain is around 23 MHz according to Equation (1). Another significant aspect of the LC sensor is the quality factor *Q*, which is calculated as

(3) $$Q=\frac{1}{R_{\mathrm{ws}}}\sqrt{\frac{L_{\mathrm{ws}}}{C_{\mathrm{ws}}}}=\frac{f_{\mathrm{res}}}{\mathrm{BW}}$$

where *BW* is the bandwidth of the resonating LC textile sensor circuit. The wireless readout performance is directly proportional to this parameter, as a sharper signal in terms of resonance frequency definition (lower bandwidth) is attainable with high *Q*. The overall resistance of the circuit has the greatest impact on *Q* with an inversely proportional relationship. The design of our wearable sensor exhibited an overall resistance (*R_WS_*) of 12 Ω. By cautiously employing selective conductive fabric materials for the capacitor and highly conductive thread for the transmission line and inductor, we achieved a relatively low *R_WS_* resulting in a quality factor of around 37 for the circuit—a comparable value to recently reported devices employing an LC circuit‐based sensing platform.^[^ ^5^, ^46^ ^]^

### 2.3 Characterization of the LC Textile Sensor Components Response to Strain

After confirming the electrical properties of the LC textile sensor components in the unstrained configuration, we further evaluated their characteristics under mechanical strain. For this, we subjected the textile components separately to strain patterns with a universal testing machine (UTM). During this process, we measured changes in the electrical parameters (*C_WS_ = C* or *L_WS_ = L*) with an inductance‐capacitance‐resistance (LCR) meter via a wired connection with the textile component (Figure S3, Supporting Information). The relatively high sampling rate of the LCR meter allows the study of impedance response of textile components at high frequencies. Specifically, the sensing metrics characterized were stress (i.e., linearity), sensitivity, drift, and stability during repetitive cycles (Experimental Section; Table S1, Supporting Information). Analogous tests were performed separately on the textile capacitor and inductor to understand the response of each component to strain.

**Figure 2:**
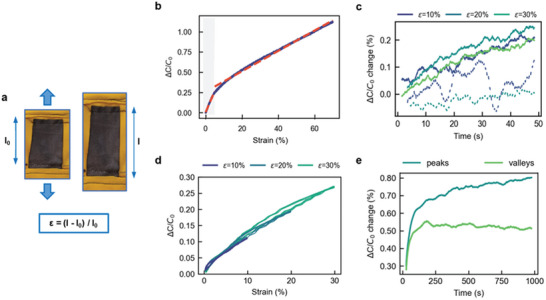
Mechanical–electrical characterization of the textile capacitor. a) Image of textile capacitor representing a uniaxial tensile test, and the expression for the calculation of strain. b) Change in capacitance of textile capacitor in response to strain. c) Drift in capacitance of textile capacitor when held at a specific strain for a duration of 50 s; solid lines represent increasing strain, dashed lines represent decreasing strain. d) Hysteresis in capacitance response for an induced strain. e) Drift in maximum (peaks) and minimum (valleys) capacitance values during long‐term stability test with imposed sinusoidal strain for 1000 cycles, when compared to the initial value.

Initially, we evaluated the electrical response of the textile capacitor with a stress–strain test (**Figure** **2**a), calculated as

(4) $$\varepsilon =\frac{l-l_{o}}{l_{o}}$$

where *I_0_* is the initial length of the textile capacitor under no strain, and *l* is the final length acquired upon strain (*ε*). The capacitance of the textile capacitor increased with strain. This behavior can be explained by the following equation that relates capacitance (of a parallel plate capacitor) to the area of the plates (*A*).

(5) $$C_{\mathrm{ws}}=\frac{\varepsilon_{o}\varepsilon_{r}A}{d}$$

where *ε*_*o*_ is the permittivity of free space, *ε*_*r*_ is the relative permittivity of the dielectric material between the plates and *d* is the distance between the plates. Upon strain, an enlarged plate area (Equation (5)) plus a reduction in the distance between the electrodes cause a rise in the overall capacitance. Here, two regions of interest were observed: first, a steeper increase in capacitance up to 5% strain (shaded area in Figure 2b), and second, above 5% strain. The gauge factor (*GF*)—or sensitivity—of the capacitive sensor can be calculated using the equation.

(6) $$\mathrm{GF}=\frac{\frac{\Delta C}{C_{0}}}{\varepsilon }$$

where *C_0_* is the capacitance under no strain and *ΔC* is the change in capacitance. The electrical response of the textile capacitor indicated a higher *GF* in the initial 5% strain region (*GF* > 4). It was previously noted that an upper limit for the strain of wearable devices could match the maximum strain of the human skin of 55%,^[^ ^47^ ^]^ and that strain sensors integrated into garments typically undergo a maximum strain of 30%.^[^ ^48^ ^]^ We hypothesize that the initial higher *GF* may be because of the imperfect distance between the conductive textile electrodes and the dielectric textile when relaxed. These layers are free to move independently and can cause a larger separation of electrodes than if they adhered. Depending on the initial relaxed state of the sensor, this phenomenon was observed to be amplified. As the textile capacitor gradually underwent further strain, the initial adjustments that occurred during the first 5–10% strain allow the textile layers to come closer. The subsequent strain of the textile capacitor thereafter delivered a linear electrical response for a strain between 5–70%. The anchoring of the sensors on the garment in a taut state resulted in accessing this broad window of the sensor's linear response.

Next, we focused on analyzing the consistency of the textile capacitive sensor. Clothing may undergo strain dynamically (i.e., during movement) or statically (i.e., sitting) and therefore—in an ideal scenario—the sensor should have a high consistency throughout different movements to achieve accurate body movement tracking. We evaluated the static consistency of our textile sensor with a step‐hold test, observing for any change in capacitance during each holding step at different strain values (Figure 2c). The variation in the capacitance over a 50 s‐hold period was negligible—less than 0.4%—which is ideal for a wearable garment meant to accurately track body movement. With the textile capacitor demonstrating a stable signal in the strain percentages expected for the wearable device, we continued to understand the dynamic response of the sensor that would occur during body movement. All materials possess some amount of mechanical hysteresis, while sensors may also possess an electrical hysteresis. As expected, the capacitive sensor presented an increasing hysteresis with increasing strain (Figure 2d; Table S2, Supporting Information), but overall showed a low value (an area of curve accounting for 4.5% of the entire area under the extended curve at 30% strain; Figure S4, Supporting Information) in comparison to other textile sensors composed of conductive composites^[^ ^40^ ^]^ or other capacitive strain sensors.^[^ ^5^ ^]^ Low hysteresis ensures predictable behavior and repeatable correlation of signal–strain during elongating or shortening and should therefore occur over repetitive cycles.

To analyze the long‐term stability of the textile capacitor, we subjected it to a repetitive strain for 1000 cycles from 10–15% strain. The capacitor displayed consistent capacitance–strain response over the 1000 cycles, with variation in the capacitance signal maxima (peaks) and the minima (valleys) within 1% of the starting values (Figure 2e). These results are encouraging for applying these sensors over a prolonged use considering that long‐term signal drifts and instabilities cause major issues when developing wearable devices. The stability of our textile capacitor is a direct result of the elasticity and consistent conductivity of textiles. Finally, wearable sensors must reliably respond to various strain rates. It has been previously reported that the range of interest for physiological movements can reach 5 Hz for a sprinter's gait.^[^ ^49^ ^]^ The capacitive sensor showed the ability to track strain without amplitude loss or signal lag up to the maximum tested frequency of 10 Hz (Figure S5a, Supporting Information).

**Figure 3:**
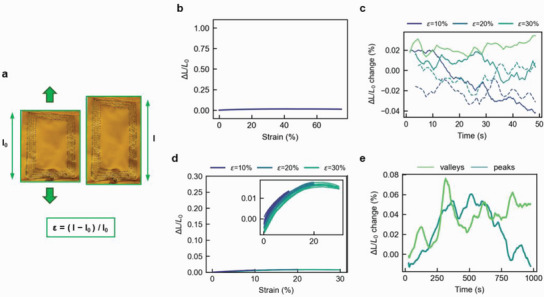
Mechanical–electrical characterization of a textile inductor. a) Image of textile inductor representing a uniaxial tensile test, and the expression for the calculation of strain. b) Change in inductance of textile inductor in response to strain. c) Drift in inductance of textile inductor when held at a specific strain for a duration of 50 s; solid lines are increasing strain, dashed lines are decreasing strain. d) Hysteresis in inductance response for an induced strain. e) Drift in maximum (peaks) and minimum (valleys) inductance values during long‐term stability test with imposed sinusoidal strain for 1000 cycles, when compared to the initial value.

With our textile capacitor performing well in all initial strain tests, we further assessed the performance of the textile inductor under a similar testing protocol to ensure that the modulation of frequency would be exclusively due to changes in capacitance and not inductance upon movement. While the characterization results indicated the textile capacitor as a suitable strain‐sensing element thanks to its increasing response to strain, the textile inductor (**Figure** **3**a) neither displayed any variation in *L* (< 4%) nor showed any correlation to strain (Figure 3b). This stable inductance is desirable for wireless readout according to our design requirements. Further, it exhibited a negligible static drift (up to 4%) during the step‐hold test (Figure 3c), low hysteresis (Figure 3d and Table S2, Supporting Information), and negligible dynamic drift (Figure 3e).

Lastly, at increasing strain rates, the textile inductor showed negligible time lag and small variation in the amplitude possibly because of the very small magnitude of inductance change (Figure S5b, Supporting Information). The electrical characteristics of the textile inductor confirmed a stable inductance value desirable for wireless readout according to our design requirements.

### 2.4 Smart Garment for Motion Tracking

**Figure 4:**
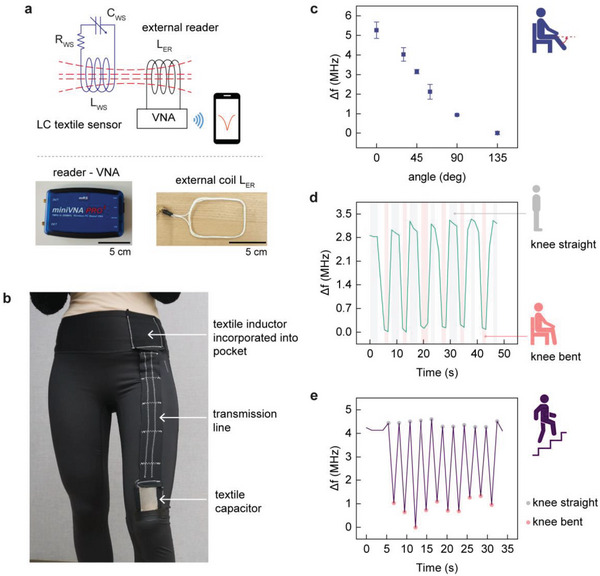
Tracking human motion with smart garment and VNA as reader. a) Scheme with pictures illustrating wireless readout of the LC textile sensor with external coil linked to VNA. b) Smart garment used for activity monitoring with LC textile sensor. Changes in resonance frequency recorded with VNA for c) different knee bending angles (data represented as the average of three independent readings with their standard deviations), d) sit‐to‐stand (squatting), and e) stair climbing activities.

After evaluating the electrical characteristics of the textile capacitor and inductor individually, we studied the resonance frequency of the combined LC sensor by coupling it with an external inductor coil connected to a VNA (**Figure** **4**a). The LC sensor was constructed by interconnecting the textile capacitor and inductor through a transmission line. Before integrating the sensor in a garment, we tracked the resonance frequency of this interconnected sensing system by determining the minimum of the reflection coefficient (*S*_11_) profile.

(7) $$S_{11}=10\mathrm{lo}g_{10}\left(\frac{P_{r}}{P_{i}}\right)$$

where *P_r_* denotes the reflected power to the reader and *P_i_* denotes the incident power on the sensor by the reader. The communication between the VNA and the wearable LC sensor was established by inductively coupling the textile inductor with an external inductor—a rectangular coil of insulated single‐strand wire with three turns—connected to the VNA. The impedance characteristics of the external reader inductor are reported in Figure S6, Supporting Information. When the reader scans frequencies by inductively coupling to the sensor, a maximum electromagnetic energy absorption by the sensor occurs at the resonance frequency indicated by a minimum value of *S_11_*. Here, the reader rectangular coil had a slightly smaller size than the textile inductor such that the two could be placed concentrically and tolerate small misalignments in the in‐plane direction. The influence of the relative position of the coupled inductors was evaluated in all three spatial directions (Figure S7a, Supporting Information). As the axial distance between the two inductors increased, the *S_11_* profile became less sharp, and its absolute value decreased progressively (Table S3, Supporting Information). A vertical displacement of 50 mm resulted in a complete signal loss (i.e., no *S_11_* change across the frequency sweep, Figure S7b, Supporting Information). The system showed tolerance to vertical displacement up to 10 mm. These data confirmed the need to maintain a small vertical distance between the two inductors, as also observed in previous studies, where the vertical separation was restricted to 2 or 2.5 mm.^[^ ^32^, ^38^ ^]^ A misalignment in the in‐plane direction of up to half of the inductor size caused complete signal loss along the longer side of the rectangular planar inductor (Figure S7c, Supporting Information) but not on the short side (Figure S7d, Supporting Information). In‐plane displacement of the two inductors seemed to have a higher impact on the frequency of the resonance peak as compared to the vertical displacement (Table S3, Supporting Information). The above inferences are in line with similar observations reported in earlier works.^[^ ^30^, ^31^ ^]^ Considering the overall influence of the relative displacement between coupled inductors on the signal quality, we chose to place the textile inductor (*L_WS_*) near the garment's pocket which would hold the reader.

To test the ability to wirelessly track human motion with our LC textile sensor, we integrated it into tight‐fitting sports pants to feature as a smart garment (Figure 4b). The textile capacitor was placed above the kneecap to track motion of the leg. The location of the textile inductor aligned with the pocket near the waist such that the external inductor coil *L_ER_* could be placed in the garment pocket adjacent to it. A suitable length of the transmission line was run vertically between the textile inductor (on the waist) and the textile capacitor (above the kneecap). Typically, the length of the transmission line influences the output signal (*S_11_*) and resonance frequency (Figure S8, Supporting Information). However, the transmission line in the present case is non‐stretchable and was sewn in a loose zigzag stitch to withstand tension, allow free movement of the leg and accommodate for slight adjustment of the line if required (Figure S9, Supporting Information).

Although downsized VNAs are inconvenient for a daily use scenario, we still tested their capability to track body movements through the response of the smart garment. We implemented the system to initially identify the knee‐bending angles by wirelessly monitoring the resonance frequency with a portable miniVNA placed in the garment's pocket. The scanned frequency range with VNA was between 10 and 30 MHz which delivered an acquisition time of 1.3 s between measurements. The bending angle was manually measured with a goniometer taking the thigh inclination as reference (illustration in Figure 4c). The resonance frequency was determined as the minimum of the *S_11_* profile for each bending angle (Figure S10, Supporting Information). We represented the response related to the knee‐bending angle (and for other tested events) in terms of change in frequency (Δ*f = f_res_ − f_min_*), that is, relative to the minimum value (*f_min_*), to recognize the typical magnitude of frequency variation occurring in each of the events. During the bending of the knee, the capacitor stretched and progressively decreased the resonance frequency from an initial value of 20.64 MHz (at 0°) to 15.38 MHz (at 135°) resulting in a Δ*f* of more than 5 MHz. The knee bending angle has been largely studied to assess the range of motion, joint function, and impact on gait. Thereby, the capability of the sensing system to detect different degrees of knee bending could potentially be applied during rehabilitation after knee surgeries^[^ ^45^ ^]^ or detection of osteoarthritis onset.^[^ ^46^ ^]^ Next, we evaluated the dynamic performance of the system for motion tracking by performing common everyday activities such as sitting‐to‐standing (equivalent to a squat exercise) and stair climbing. The response of the system revealed the ability to track these motions with a maximum Δ*f* of 3.06 and 3.55 MHz for sitting‐to‐standing and stair‐climbing activities, respectively (Figure 4d,e). We further evaluated the influence of the zig‐zag stitched transmission line on the frequency response by stretching the ends of the anchoring cloth and thereby forcing the transmission line to adjust in the process. The *S_11_* curves obtained with VNA during these experiments revealed a nearly constant resonance frequency for a stretch of at least 3 cm (corresponds to 50% strain with textile capacitor), with a maximum shift of 16 kHz (Figure S11, Supporting Information). We also assessed the performance of the sensor after repeated washing and in real‐life environmental conditions (Figure S12, Supporting Information). After repeated washing with water (at least 10 times), the sensor showed Δ*f* of around 4.51 ± 0.21 MHz for a 30% strain, which is close to the initial value (before washing) of 4.48 MHz. The sensor also showed a stable response to changes in relative humidity (between 20% *RH* and 75% *RH*) and temperature (between 15 and 40 °C) with an Δ*f* of 4.61 ± 0.13 and 4.75 ± 0.10 MHz, respectively—again compared to the initial value of 4.48 MHz.

The use of a VNA is ideal for characterization, but they are not suitable as a real‐time communication device for wearable systems as they are bulky and slow to read. We had to deliberately perform the above‐detailed movements at a slower pace to register the frequency changes with the miniVNA. Therefore, we sought to design and employ a custom wireless readout circuit that would allow faster data acquisition with a smartphone at sampling rates suitable for motion‐tracking with wearable devices.

### 2.5 fReader for Motion Tracking with the Smart Garment

**Figure 5:**
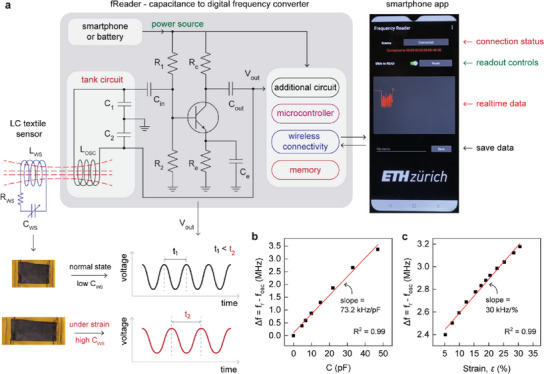
fReader wireless reading module. a) Scheme illustrating the construction of fReader and details of the workflow for wirelessly reading out the LC textile sensor response. The coil of the fReader inductively couples with the textile inductor and converts the strain‐induced capacitance changes of the textile capacitor to a digital frequency that can be read out with the custom‐built application over a smartphone. b) Changes in frequency registered by the fReader with commercial capacitors, where *f_r_* and *f_osc_* correspond to frequency obtained at *C* = 0 pF and at capacitance *C*. c) Changes in frequency registered by the fReader with textile capacitor under different strains, where *f_osc_* correspond to the frequency obtained at a particular strain.

To simplify the readout approach for faster data acquisition and to construct a reader in a convenient form factor suitable for motion‐tracking with garments, we designed a lightweight compact reader that can comfortably sit in a garment's pocket; the circuit was constructed using easily available off‐the‐shelf components and a case was 3D printed to shield the circuit and expose only the fReader's inductor coil as shown in Figure 1. When placed in the smart garment's pocket (of similar configuration as shown in Figure 4b), the fReader coil inductively couples with the textile inductor on the pants. The fReader reports the capacitance of the textile capacitor in the form of an oscillating voltage signal (*V_out_*) of a specific frequency (*f_osc_*). As illustrated in **Figure** **5**a, the frequency of this oscillating signal changes based on the state of the textile capacitor. Under no strain, *C_WS_* is low and the oscillator outputs a high‐frequency signal (*f_1_*). In comparison, the output frequency would decrease (*f_2_ < f_1_*) when the textile capacitor is under strain; this is due to a higher value of *C_WS_* resulting in a greater overall load capacitance *C_L_* for the oscillator (Equation (2)). The microcontroller in the reader realizes a digital frequency value of the oscillating signal by analyzing the number of cycles during a fixed gated time. Further, the reader establishes a wireless connection with a custom‐designed smartphone application and has the capability to record and store digital frequency readings. The fReader draws the operating power (625 mW) from a dedicated battery or from the smartphone itself.

We initially evaluated the fReader sensitivity by coupling it with a textile inductor connected to commercial capacitors. This experiment served to measure the fReader's response over a broad capacitance range including the textile capacitor's values acquired during strain. For this, we used a textile inductor and a commercial inductor (on the fReader) of nearly an equivalent inductance value (Figures S13 and S14, Supporting Information). It is expected that the oscillation frequency *f_osc_* decreases with an increase in capacitance as indicated by Equation (2). So, to acquire a relative magnitude, we considered here the oscillation frequency *f_r_* obtained with no capacitor connected across the textile inductor as a reference value. As indicated in Figure 5b, the changes in oscillation frequencies (Δ*f = f_r_ − f_osc_*) of the fReader showed a linear relationship for capacitance changes up to at least 50 pF, offering a sensitivity of 73.2 kHz per pF. In a similar way, Δ*f* varied linearly with strain when examined with the textile capacitor subjected to strain using a UTM. Considering the typical maximum strain with textiles during movement together with the sensor's taut state on the garment, the characteristic operation region corresponds to around 5–30% strain. The sensing system with fReader delivered a sensitivity of 30 kHz per % with a mean absolute percentage error of 0.7% (Figure 5c) and indicated the ability to track strain changes below 1% in this region (Figure S15, Supporting Information). Further, the fReader was able to track changes in capacitance signal at an imposed strain frequency of up to 10 Hz without any loss in amplitude (Figure S16, Supporting Information).

**Figure 6:**
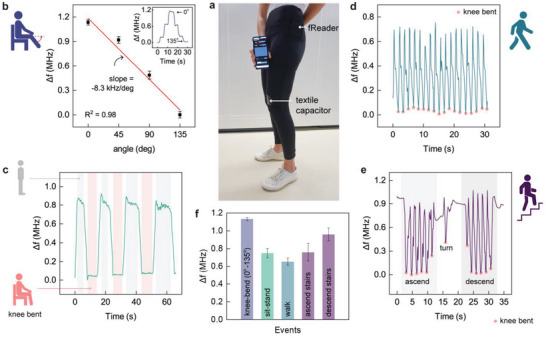
Tracking human movements with smart garments and fReader. a) The picture shows the smart garment with an integrated LC textile sensor, alongside the fReader in the pant pocket to inductively couple with the textile inductor on the garment. The fReader tracks strain‐induced capacitive changes in the textile capacitor through inductive coupling and wirelessly transmits this information to a custom‐designed application in a smartphone. b–f) Variation in the frequencies registered with the combined system of the LC sensor featured smart garment and fReader, for different movements in daily activities like knee‐bending, sitting‐standing, walking, and stair climbing. The data in (b,f) are represented as an average value of three independent readings with standard deviation.

With a functioning system, we moved on to track body movements observed in day‐to‐day activities by placing the fReader in the pocket of our smart garment as shown in **Figure** **6**a. For these experiments, we set the acquisition time between readings to 150 ms—distinct improvement versus the capability of the portable VNA with an acquisition time of 1.3 s. To recognize the typical magnitude of frequency variation occurring in each of the tested events, we reported frequency‐related information in terms of change in frequency (Δ*f = f_osc_ − f_min_*), that is, relative to the minimum value (*f_min_*). For a range of angular knee movements performed while sitting (Figure 6b), the smart garment–fReader system had a sensitivity of 8.3 kHz per degree under a linear‐fit approximation and was able to clearly distinguish the movements related to activities like sitting and standing (Figure 6c). The smartphone application further allowed observing the LC sensor response related to human motion occurring at a natural pace in contrast to the earlier‐mentioned deliberate slow‐paced movements with VNA. A real‐time demonstration with an event like walking (Figure 6d) is shown in Video S1, Supporting Information; the textile capacitor responded to movements (indicated through frequency changes) and the fReader registered the strain‐induced capacitance changes when positioned in the pocket. In fact, the fReader was able to run with the power delivered from the smartphone itself while both were residing in the pocket. Since the construction and operation principle of fReader (Figure 5) is simple compared to VNAs or spectrum analyzers and built with inexpensive and easily available components, it opens the possibility to integrate it into a smartphone case—or better—directly as part of the hardware of the smartphone itself. The fReader was also able to register frequency changes induced by the textile capacitor while ascending and descending the stairs (Figure 6e). Overall, the maximum Δ*f* obtained in all the above‐monitored events is beyond 0.6 MHz (Figure 6f); while the events of sitting‐standing, walking, and stair ascending‐descending fall within the indicated frequency change margin of the angular knee‐bends.

**Figure 7:**
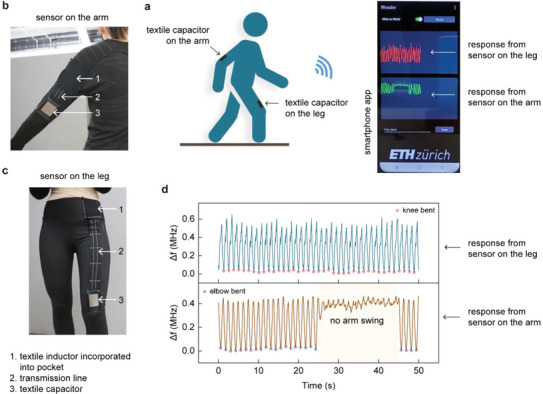
Tracking human movements at multiple locations with smart garment and fReader. a) Scheme depicting the locations of the textile capacitors for tracking movement of the leg and arm while walking. A custom‐designed smartphone app wirelessly communicates with the fReader modules to instruct and receive responses from the individual sensors. b,c) Pictures of the smart garments with the LC textile sensors. d) Variation in the frequencies obtained with the wireless sensing system for movement of the leg and arm during walking.

As a demonstration of the concept to track different body movements, we further integrated the LC textile sensor at various locations on the smart garment. In addition to the earlier‐mentioned LC textile sensor above the kneecap, we placed a second LC textile sensor on a long‐sleeve sport shirt (above the elbow) to track both leg and arm movement simultaneously during walking, as depicted in **Figure** **7**a. The fReader coupled with the LC textile sensor records the sensing information and communicates it wirelessly to a custom‐developed smartphone app. First, we studied the typical frequency variation during the bending of the arm with a second LC textile sensor (Figure 7b) and the fReader. The LC textile sensor in the sport shirt responded to typical arm movements between 0° and 135° through strain‐induced capacitance changes of the textile capacitor, with a sensitivity of 3.5 kHz per degree under a linear‐fit approximation (Figure S17, Supporting Information). With both the sensors integrated into the smart garment (Figure 7b,c), we then simultaneously tracked the leg and arm movements during walking (Video S2, Supporting Information). The smartphone app displayed the frequency responses from both the LC textile sensors, with a typical decrease in frequency upon bending of the leg or the arm (Figure 7d). Although we demonstrated the possibility of using the sensing system at two different sensing nodes, the number of nodes can be increased based on the desired application.

## 3 Conclusion

In this work, we demonstrated a sensing approach that allowed a garment in a pure textile form to track body movements and wirelessly transmit motion‐related information. The system developed with this approach accomplished the task by incorporating a passive LC textile sensor in a smart garment and wirelessly retrieving the sensor response with a custom‐built reader and smartphone application. The strain‐induced capacitance changes of the LC wearable sensor during body movements modulated the frequency response in the reader enabling tracking of movement. The complete absence of rigid components on the garment is a key advancement in eliminating all commercial silicon‐based sensor tags on the skin or on the clothing. This achievement with minimalistic components (only textile inductor and capacitor) opens the possibility for a truly unobtrusive integration of sensing functionality in everyday clothing. By integrating the LC textile sensor into the garment, we demonstrated the ability to track routine daily activities. Moreover, the sensing system is sewing‐friendly and may be easily placed at different locations on the garment with little adaptation and presents the concrete potential to be streamlined in standard clothing manufacturing.

To overcome the limitation of bulky and expensive VNAs towards wearable applications, we developed a lightweight, low‐cost fReader in a convenient form factor to enable wireless readout of the LC textile sensor. The fReader provided higher sampling rates than downsized/portable VNAs to track body movement occurring at a natural pace with the LC textile sensor. It provided a real‐time response by wirelessly communicating with a smartphone creating opportunities for more accessible motion tracking to aid areas of rehabilitation, injury prevention, and athletic performance improvement. The fReader can further operate through power delivered from the smartphone and comfortably be integrated as a case enhancing the user‐friendliness, acceptance, and accessibility of the system. Given the reduced complexity when compared to VNAs, the simple circuit design of fReader built with easily available components creates the possibility for future integration into the smartphone hardware itself. Moreover, unlike other wireless readout approaches with capacitive textile sensors for activity tracking, the developed sensing approach requires no physical connections of the LC textile sensor with silicon chips or other rigid components. Overall, the robust system described here may create possibilities for novel passive wearables to monitor movement wirelessly, particularly in complex situations where it becomes impractical to employ alternative available technologies.

## 4 Experimental Section

### Materials and Instrumentation

Electrolycra 234–309A was purchased from MindSets (Saffron Walden, UK). Yellow spandex textile with one‐way stretch was purchased from Spitzenraum (Schänis, Switzerland). The Liberator thread was purchased from Syscom Advanced Materials (Columbus, OH, United States). All electronic components were purchased from Digikey (Switzerland) and Distrelec (Switzerland). Sewing was completed on a Singer Heavy Duty machine (Model 4423), whereas embroidery was carried out with a Brother Innov'is 2600. Conductivity measurements were completed with a digital multimeter (Fluke 177, Everett, Washington, USA). Impedance measurements of the unstretched samples were performed with a HIOKI IM7581‐1 impedance analyzer (Ueda, Nagano, Japan). Tensile tests were accomplished with the Instron E3000 universal tensile measurement (UTM) device (Norwood, MA, United States). Impedance measurements during the tensile tests were performed with a precision LCR meter—Hioki IM3536 LCR (Ueda, Nagano, Japan) with a four‐lead probe setup. For tensile–electronic measurements, the LCR meter was interfaced with a PC (using a custom‐developed LabView script) and synchronized with the UTM using a Data Acquisition module (National Instruments, Austin, TX, US). Vector Network Analyser measurements were completed using a miniVNA pro (MiniRadioSolutions, Herxheim, Germany) and a planar rectangular inductor coil (80 × 60 mm, 3 turns) built with an insulated copper wire (22G).

### Fabrication of Smart Garment with Textile Capacitor and Inductor

The capacitive sensor was arranged in a double plate configuration by stitching two conductive stretchable textiles of dimensions 50 × 35 mm on either side of a double‐layered yellow spandex textile (Figure 1a, *C_WS_*). The electrodes were sewn to the base yellow spandex in a taut configuration—to avoid wrinkling and precisely overlap in area. Care was taken to avoid shorting between the two electrodes during sewing. The DC resistance of the electrodes obtained with multiple samples resulted in a mean value of 1.0 Ω cm^−1^ (from the manufacturer: 0.5 Ω cm^−1^ when unstretched, 1.33 Ω cm^−1^ when stretched in one axis, and 0.16 Ω cm^−1^ when stretched at 90° to this).

The textile inductor used for the tests with the VNA was manufactured by sewing conductive Liberator thread on a piece of spandex fabric with a standard sewing machine. The thread was sewn in a rectangular loop of dimensions 80 × 60 mm, with four turns and a 2 mm gap between the turns (Figure 3a). A consistent gap between the loops was ensured during fabrication. A wash‐away sewing stabilizer was used to avoid wrinkles and produce a flat textile inductor. A highly conductive and mechanically stiff thread was used to minimize resistance and ensure mechanical robustness. The measured DC resistance of a 1 cm long thread was typically 0.036 Ω cm^−1^ (3.3 Ω m^−1^ according to the manufacturer). The textile inductor used for the tests with the fReader was manufactured by embroidering Liberator thread on a piece of spandex fabric with a standard embroidery machine. The thread was stitched in a rectangular loop of dimensions 55 × 45 mm, with six turns and a 1 mm gap between the turns (Figure 1a, *L_WS_*).

The textile capacitive and textile inductor were connected to form the LC textile sensor with the same conductive thread used to manufacture the inductor. First, this thread was attached to each of the two electrodes of the capacitor. Of note, the textile inductor was sewn adjacent to the smart garment´s pocket and in an anatomical location where limited strain would take place. The textile capacitor was intentionally placed above the kneecap to minimize out‐of‐plane deformation (i.e., compression of the two plates against each other). This arrangement allowed the capacitor to undergo in‐plane deformation from the stretching of the plates when flexing the knee.

### Mechanical Characterization

The textile components were tested to determine their response to strain with a UTM at a strain sampling rate of 100 Hz by applying a linear displacement to the samples (no torsional displacement). The UTM was used with custom‐made 3D printed fixtures to conveniently fix the textile sample through shafts going through custom‐made sewn loops (Figure S3, Supporting Information). The UTM load was calibrated at the beginning of each test. The UTM was programmed to execute pre‐defined displacement profiles, with displacements scaled to the textile sample sizes to ensure consistent strain across the samples. The strain was calculated from raw displacement “*l* ” using taut length according to Equation (4); sensor strain was determined for the complete textile sensor (electrodes/dielectric separator), where *l_0_* and *A_0_* denote the unstrained sensor length and area, respectively. In case of textile capacitor, the area of the electrodes was used as *A_0_*. The maximum values of strain for both samples were calculated based on the actual measured length at the maximum displacement attainable with the UTM.

The electrical behavior of the sensors during the tests was acquired with a precision LCR meter with a four‐lead probe setup. The electrically conductive thread terminals of the samples were clipped to the LCR meter probes. The details of various mechanical tests conducted on textile samples (Table S1, Supporting Information) are given below.

### Tensile Stress–Strain Test

This test was conducted by subjecting the textile sample between 0% and maximum attainable strain corresponding to the maximum stroke of the machine head (70% for the capacitor, 80% for the inductor) at a strain rate of 1% per second. The Young's modulus of the samples was calculated as the slope of the linearly fitted data. Similarly, the linearity of the electrical response and the GF were obtained from this test.

### Step‐Hold (Static Drift) Test

This test was conducted by subjecting the textile sample to a step‐hold strain pattern in three steps, inducing a 10% strain in each step in the range of 0% to 30% strain at a strain rate of 1% per second. Here, the ramp‐up and ramp‐down phases (*t_ramp_*) were 10 s long and the hold phase (*t_hold_*) was 50 s long. The variation in the electrical response during the hold phase was evaluated as variation with respect to the baseline (first value of the hold phase).

### Stress‐Release (Hysteresis) Test

This test was conducted by subjecting the textile sample to a stress pattern of three sets of triangular waves. Each set contained three cycles of triangular waves accounting for the maximum strain of 10%, 20%, and 30% at a strain rate of 1% per second. This process resulted in a closed loop curve made up of forward (extension) and backward (relaxation) curves. An ideal sensor with no hysteresis gave the same response when extended and relaxed. Hysteresis was defined as the area enclosed in the closed curves (extension‐relaxation). Further, hysteresis was also evaluated as the ratio between the area enclosed in the extension‐relaxation loop divided by the area under the curve of the extension curve (Figure S4, Supporting Information).

### Long‐Term Stability (Dynamic Drift) Test

This test was conducted by subjecting the textile sample to a stress pattern of 1000 sinusoidal waves (*f = 1 Hz*) inducing a stress of about 5% on a 10% pre‐strained sample. The magnitude of variation in peaks and valleys of the electrical response, when compared to the values obtained in the first strain cycle, was used as a metric.

### Bandwidth Test

This test was conducted by subjecting the textile sample to a strain pattern of 30 sinusoidal waves of fixed amplitude and increasing frequencies. The ability of the textile component to track the strain sinusoidal wave without amplitude loss or strain–electrical signal lag was used as the evaluation metric. This was assessed by an analysis in the frequency domain (Figure S5, Supporting Information). It was worth noting the test equipment limitation: the UTM was not able to supply sinusoidal waves of consistent amplitude for higher frequencies such as typically above 5 Hz during the initial and final cycles. In such cases, those cycles were excluded and considered the sinusoidal waves of consistent frequency.

### Susceptibility to Environmental Conditions—Washability Test

The pants were washed in cold water, dabbed with a towel, and let to dry completely at room temperature. The resonance frequency was measured with the miniVNA after the first three washes and after the tenth wash in the unstrained and strained (*ε* = 30%) configuration.

### Susceptibility to Environmental Conditions—Humidity Test

The influence of humidity was assessed in naturalistic conditions in two different environments: in the authors’ laboratory (22 °C and 20% *RH*), and Botanical Gardens of the University of Zurich (22 °C and 75% *RH*). The temperature and humidity readings were acquired using Testo 635‐1 (Testo, Thailand). The resonance frequency in strained and unstrained configurations was measured with the miniVNA.

## Conflict of Interest

T.C., V.G., C.A., and C.M. are co‐inventors on a patent application that is based on this research.

## Author Contributions

V.G. and S.K.S contributed equally to this work. C.M., T.C., C.A., S.K.S., and V.G. contributed to the conception and design of the study. V.G. fabricated the textile samples and prototype. S.K.S. developed the wireless reader modules. V.G. and S.K.S. ran the experiments, analyzed the data, and prepared graphical material. V.G., S.K.S., and T.C. wrote the initial draft of the manuscript. B.H. contributed to the wireless reading tests with VNA and mechanical‐electrical tests. All authors contributed to revising the manuscript.

## Supporting information

Supporting Information

Supplemental Video 1

Supplemental Video 2

## Data Availability

The data that support the findings of this study are available in the supplementary material of this article.

## References

[1] S. S. Gambhir , T. J. Ge , O. Vermesh , R. Spitler , G. E. Gold , Sci. Transl. Med. 2021, 13, abe5383. 10.1126/scitranslmed.abe5383 34108250

[2] J. Kim , A. S. Campbell , B. E. F. de Ávila , J. Wang , Nat. Biotechnol. 2019, 37, 389. 30804534 10.1038/s41587-019-0045-y PMC8183422

[3] J. M. Peake , G. Kerr , J. P. Sullivan , Front. Physiol. 2018, 9, 743. 30002629 10.3389/fphys.2018.00743 PMC6031746

[4] S. R. Steinhubl , E. D. Muse , E. J. Topol , Sci. Transl. Med. 2015, 7, 283rv3. 10.1126/scitranslmed.aaa3487 PMC4748838 25877894

[5] J. Lee , S. J. Ihle , G. S. Pellegrino , H. Kim , J. Yea , C. Y. Jeon , H. C. Son , C. Jin , D. Eberli , F. Schmid , B. L. Zambrano , A. F. Renz , C. Forró , H. Choi , K. I. Jang , R. Küng , J. Vörös , Nat. Electron. 2021, 4, 291.

[6] R. Herbert , H.‐R. Lim , S. Park , J.‐H. Kim , W.‐H. Yeo , Adv. Healthcare Mater. 2021, 10, 2100158. 10.1002/adhm.202100158 34019731

[7] T. Q. Trung , N.‐E. Lee , Adv. Mater. 2016, 28, 4338. 26840387 10.1002/adma.201504244

[8] Y. Khan , A. E. Ostfeld , C. M. Lochner , A. Pierre , A. C. Arias , Adv. Mater. 2016, 28, 4373. 26867696 10.1002/adma.201504366

[9] J. Wang , C. Lu , K. Zhang , Energy Environ. Mater. 2020, 3, 80.

[10] H. C. Koydemir , A. Ozcan , Annu. Rev. Anal. Chem. 2018, 11, 127. 10.1146/annurev-anchem-061417-125956 29490190

[11] S. Lee , S. Gandla , M. Naqi , U. Jung , H. Youn , D. Pyun , Y. Rhee , S. Kang , H.‐J. Kwon , H. Kim , M. G. Lee , S. Kim , IEEE Trans. Ind. Electron. 2020, 67, 8808.

[12] D. Son , J. Kang , O. Vardoulis , Y. Kim , N. Matsuhisa , J. Y. Oh , J. W. To , J. Mun , T. Katsumata , Y. Liu , A. F. McGuire , M. Krason , F. Molina‐Lopez , J. Ham , U. Kraft , Y. Lee , Y. Yun , J. B.‐H. Tok , Z. Bao , Nat. Nanotechnol. 2018, 13, 1057. 30127474 10.1038/s41565-018-0244-6

[13] J. Park , J. Kim , S.‐Y. Kim , W. H. Cheong , J. Jang , Y.‐G. Park , K. Na , Y.‐T. Kim , J. H. Heo , C. Y. Lee , J. H. Lee , F. Bien , J.‐U. Park , Sci. Adv. 2018, 4, aap9841. 10.1126/sciadv.aap9841 PMC5787380 29387797

[14] M. S. Mannoor , H. Tao , J. D. Clayton , A. Sengupta , D. L. Kaplan , R. R. Naik , N. Verma , F. G. Omenetto , M. C. McAlpine , Nat. Commun. 2012, 3, 763. 22453836 10.1038/ncomms1767

[15] I. Wicaksono , C. I. Tucker , T. Sun , C. A. Guerrero , C. Liu , W. M. Woo , E. J. Pence , C. Dagdeviren , npj Flexible Electron. 2020, 4, 5. 10.1038/s41528-020-0068-y PMC7222957 38624354

[16] N. A. Choudhry , L. Arnold , A. Rasheed , I. A. Khan , L. Wang , Adv. Eng. Mater. 2021.

[17] T. Wu , F. Wu , J.‐M. Redoute , M. R. Yuce , IEEE Access 2017, 5, 11413.

[18] X. Tao , T.‐H. Huang , C.‐L. Shen , Y.‐C. Ko , G.‐T. Jou , V. Koncar , Adv. Mater. Technol. 2018, 3, 1700309.

[19] X. Tian , P. M. Lee , Y. J. Tan , T. L. Y. Wu , H. Yao , M. Zhang , Z. Li , K. A. Ng , B. C. K. Tee , J. S. Ho , Nat. Electron. 2019, 2, 243.

[20] Y. S. Oh , J. H. Kim , Z. Xie , S. Cho , H. Han , S. W. Jeon , M. Park , M. Namkoong , R. Avila , Z. Song , S. U. Lee , K. Ko , J. Lee , J. S. Lee , W. G. Min , B. J. Lee , M. Choi , H. U. Chung , J. Kim , M. Han , J. Koo , Y. S. Choi , S. S. Kwak , S. B. Kim , J. Kim , J. Choi , C. M. Kang , J. U. Kim , K. Kwon , S. M. Won , et al., Nat. Commun. 2021, 12, 5008. 34429436 10.1038/s41467-021-25324-w PMC8385057

[21] M. H. Kang , G. J. Lee , J. H. Yun , Y. M. Song , Sensors 2021, 21, 878. 33525509 10.3390/s21030878 PMC7865650

[22] Lin, R. L. , H. J. Kim , S. Achavananthadith , S. A. Kurt , S. C. C. Tan , H. Yao , B. C. K. Tee , J. K. W. Lee , J. S. Ho , Nat. Res. 2020, 11, 444. 10.1038/s41467-020-14311-2 PMC6978350 31974376

[23] S. Han , J. Kim , S. M. Won , Y. Ma , D. Kang , Z. Xie , K.‐T. Lee , H. U. Chung , A. Banks , S. Min , S. Y. Heo , C. R. Davies , J. W. Lee , C.‐H. Lee , B. H. Kim , K. Li , Y. Zhou , C. Wei , X. Feng , Y. Huang , J. A. Rogers , Sci. Transl. Med. 2018, 10, aan4950. 10.1126/scitranslmed.aan4950 PMC5996377 29618561

[24] A. Hajiaghajani , A. H. Afandizadeh Zargari , M. Dautta , A. Jimenez , F. Kurdahi , P. Tseng , Nat. Electron. 2021, 4, 808.

[25] S. Niu , N. Matsuhisa , L. Beker , J. Li , S. Wang , J. Wang , Y. Jiang , X. Yan , Y. Yun , W. Burnett , A. S. Y. Poon , J. B. H. Tok , X. Chen , Z. Bao , Nat. Electron. 2019, 2, 361.

[26] T. Lee , W. Lee , S.‐W. Kim , J. J. Kim , B.‐S. Kim , Adv. Funct. Mater. 2016, 26, 6206.

[27] J. Lee , H. Kwon , J. Seo , S. Shin , J. H. Koo , C. Pang , S. Son , J. H. Kim , Y. H. Jang , D. E. Kim , T. Lee , Adv. Mater. 2015, 27, 2433. 25692572 10.1002/adma.201500009

[28] S. K. Behera , IEEE Sens. J. 2022, 22, 1105.

[29] B. Garnier , P. Mariage , F. Rault , C. Cochrane , V. Koncar , Sci. Rep. 2021, 11, 2159. 33495482 10.1038/s41598-021-81246-z PMC7835243

[30] S. Micus , L. Padani , M. Haupt , G. T. Gresser , Appl. Sci. 2021, 11, 4309.

[31] A. Hajiaghajani , P. Tseng , Adv. Mater. Technol. 2021, 6, 4.

[32] P. Tseng , B. Napier , L. Garbarini , D. L. Kaplan , F. G. Omenetto , Adv. Mater. 2018, 30, 1703257. 10.1002/adma.201703257 29572979

[33] J. Kim , M. Kim , M. S. Lee , K. Kim , S. Ji , Y. T. Kim , J. Park , K. Na , K. H. Bae , H. K. Kim , F. Bien , C. Y. Lee , J. U. Park , Nat. Commun. 2017, 8, 14997. 28447604 10.1038/ncomms14997 PMC5414034

[34] L. Ma , R. Wu , A. Patil , S. Zhu , Z. Meng , H. Meng , C. Hou , Y. Zhang , Q. Liu , R. Yu , J. Wang , N. Lin , X. Y. Liu , Adv. Funct. Mater. 2019, 29, 1904549.

[35] R. Wu , L. Ma , A. Patil , C. Hou , S. Zhu , X. Fan , H. Lin , W. Yu , W. Guo , X. Y. Liu , ACS Appl. Mater. Interfaces 2019, 11, 33336. 31424911 10.1021/acsami.9b10928

[36] J. S. Heo , J. Eom , Y.‐H. Kim , S. K. Park , Small 2018, 14, 1703034.

[37] W. Zeng , L. Shu , Q. Li , S. Chen , F. Wang , X. M. Tao , Adv. Mater. 2014, 26, 5310. 24943999 10.1002/adma.201400633

[38] Y. Wang , Q. Tan , L. Zhang , B. Lin , M. Li , Z. Fan , Micromachines 2021, 12, 34. 10.3390/mi12010034 PMC7823390 33396867

[39] M. Demori , M. Baù , M. Ferrari , V. Ferrari , Micromachines 2018, 9, 449. 30424382 10.3390/mi9090449 PMC6187290

[40] B. C. Hannigan , T. J. Cuthbert , W. Geng , M. Tavassolian , C. Menon , Front. Mater. 2021, 8, 639823.

[41] J. Choi , K. Hong , Appl. Ergon. 2015, 48, 186. 25683546 10.1016/j.apergo.2014.11.016

[42] M. Totaro , T. Poliero , A. Mondini , C. Lucarotti , G. Cairoli , J. Ortiz , L. Beccai , Sensors 2017, 17, 2314. 29023365 10.3390/s17102314 PMC5677432

[43] A. Atalay , V. Sanchez , O. Atalay , D. M. Vogt , F. Haufe , R. J. Wood , C. J. Walsh , Adv. Mater. Technol. 2017, 2, 1700136.

[44] M. Tavassolian , T. J. Cuthbert , C. Napier , J. Peng , C. Menon , Adv. Intell. Syst. 2020, 2, 1900165.

[45] A. G. Patiño , C. Menon , Sensors 2021, 21, 225.33401380

[46] B. Nie , R. Huang , T. Yao , Y. Zhang , Y. Miao , C. Liu , J. Liu , X. Chen , Adv. Funct. Mater. 2019, 29, 1808786.

[47] T. Yamada , Y. Hayamizu , Y. Yamamoto , Y. Yomogida , A. Izadi‐Najafabadi , D. N. Futaba , K. Hata , Nat. Nanotechnol. 2011, 6, 296. 21441912 10.1038/nnano.2011.36

[48] W. Geng , T. J. Cuthbert , C. Menon , ACS Appl. Polym. Mater. 2021, 3, 122.

[49] A. I. T. Salo , I. N. Bezodis , A. M. Batterham , D. G. Kerwin , Med. Sci. Sports Exercise 2011, 43, 1055. 10.1249/MSS.0b013e318201f6f8 20980924

