# Supplementary material for: Passive and Wireless All‐Textile Wearable Sensor System
Source: Adv Sci (Weinh). 2023 May 19;10(22):2206665. doi: 10.1002/advs.202206665 (PMC10401120; doi:10.1002/advs.202206665)
Supplement: Supplementary file 1 — Supporting Information [file ADVS-10-2206665-s002.pdf]

ADVANCED SCIENCE
Open Access

Supporting Information

for *Adv. Sci.*, DOI 10.1002/advs.202206665

Passive and Wireless All-Textile Wearable Sensor System

Valeria Galli, Sunil Kumar Sailapu, Tyler J. Cuthbert, Chakaveh Ahmadizadeh, Brett C. Hannigan and Carlo Menon**

Supporting Information

Passive and Wireless All-Textile Wearable Sensor System

Valeria Galli,[1,a] Sunil Kumar Sailapu,[1,a] Tyler J. Cuthbert,[1,*] Chakaveh Ahmadizadeh,[1] Brett C. Hannigan,[1] Carlo Menon[1,*]

[1]Biomedical and Mobile Health Technology (BMHT) Group, ETH Zürich, Zürich, Switzerland.

[a]the authors contributed equally

*correspondence: tyler.cuthbert@hest.ethz.ch, carlo.menon@hest.ethz.ch

Supporting tables

Table S1. **Protocol for characterization of the samples' response to mechanical strain**.

**Test**	**Description**	**Derived result**	**Time – displacement profile**
Stress-strain	• 1 cycle<br>• Linear<br>• ε = 0 – 70% for C_{ws} *<br>• ε = 0 – 80% for L_{ws} **<br>• $\dot{\varepsilon} = \frac{1\%}{s}$	elastic modulus, linear range, gauge factor (sensitivity)	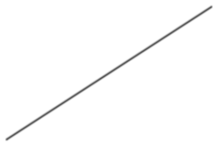
Step-hold	• 1 cycle<br>• Linear<br>• ε = 10%, 20%, 30%<br>• $\dot{\varepsilon} = \frac{1\%}{s}$<br>• t_{ramp} = 10 s, t_{hold} = 50 s	static drift	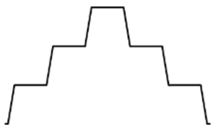
Stress-release	• 3 cycles<br>• Triangular<br>• ε = 10%, 20%, 30%<br>• $\dot{\varepsilon} = \frac{1\%}{s}$	hysteresis	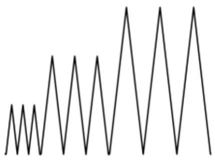
Long term stability	• 1000 cycles<br>• Sinusoidal<br>• ε_0 = 10%<br>• ε = 5%<br>• f = 1 Hz	dynamic drift, stability	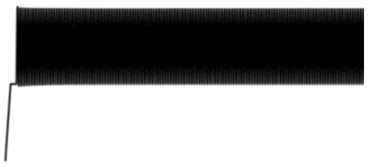
Bandwidth	• 30 cycles<br>• sinusoidal<br>• f = 0.1 Hz, 1 Hz, 2 Hz, 5 Hz, 10 Hz	sensor response to frequency of applied strain	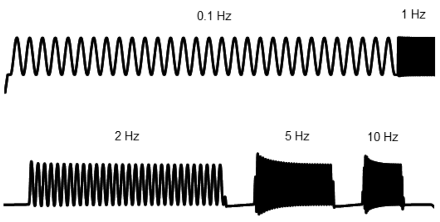

ε = strain; ε_0 = pre-strain; $\dot{\varepsilon} = \frac{d\varepsilon}{dt}$ = strain rate.; t_{ramp} = ramp up and ramp down time; t_{hold} = hold time for each step

* For the textile capacitive sensor (C_{WS}), we experimentally observed that the actual length of the sample at the maximum displacement provided by the universal testing machine (UTM) was l = 85 mm = 1.7 l_0, which corresponds to ε = 70%.

** For the textile inductor (L_{WS}), the maximum strain was limited by the maximum stroke that the UTM could provide (60 mm) in relation to the inductor size (80 mm).

Table S2. Mechanical hysteresis tests. Hysteresis values (mean±standard deviation over three cycles) calculated as the area enclosed by the curves (strain-impedance variation) normalized by the area under the curve of the extension phase.

**Textile capacitor (C_{WS})**		**Textile inductor (L_{WS})**	
**ε**	**Hysteresis area (%)**	**ε**	**Hysteresis area (%)**
10%	1.68 ± 0.10	10%	9.91 ± 2.92
20%	3.51 ± 1.17	20%	4.82 ± 0.83
30%	4.48 ± 0.38	30%	1.91 ± 0.67

Table S3. Influence of reciprocal displacement between sensor inductor and reader inductor.

		**f_{res} (MHz)**	**$\|S_{11}\|$ (dB)**
**x**	a/8	23.34	21.82
	a/4	25.74	12.83
	a/2	21.90	1.40
**y**	b/8	22.81	10.48
	b/4	25.15	9.49
	b/2	21.63	3.41
**z**	5 mm	23.23	16.95
	10 mm	22.38	9.24
	25 mm	21.42	1.19
	50 mm	20.94	0.41

Supporting figures

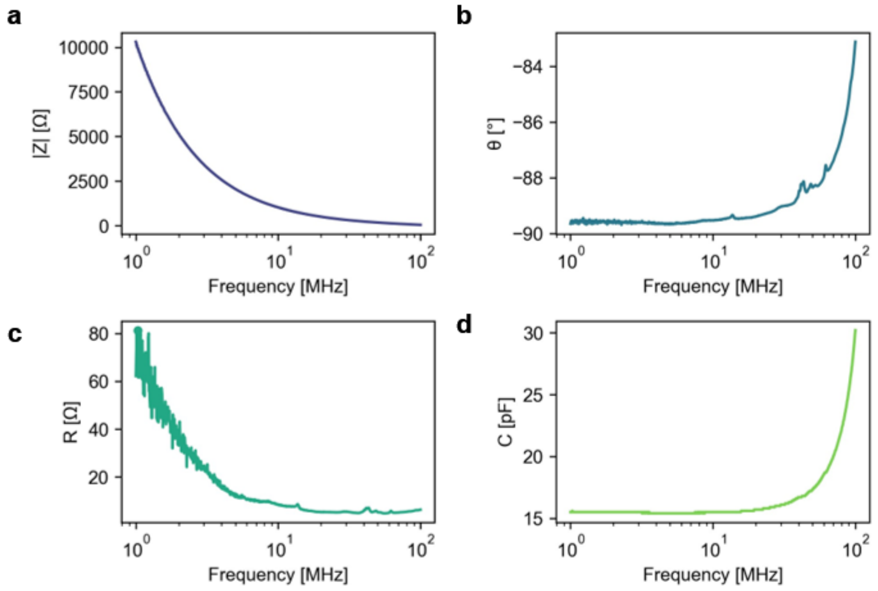

Figure S1. **Impedance characterization of the textile capacitor**. a,b) Magnitude and phase of textile capacitor's impedance. The textile capacitor displays capacitive behaviour with -88° < θ < -90° until f = 60 MHz. c,d) Magnitude of the resistive and capacitive component of the textile capacitor.

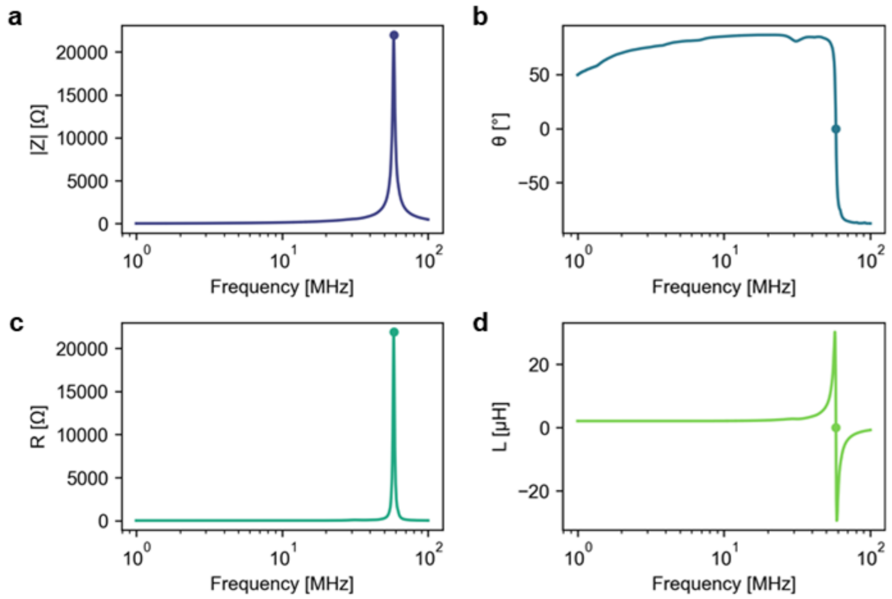

Figure S2. Impedance characterization of the textile inductor. a,b) Magnitude and phase of textile inductor's impedance. c,d) Magnitude of the resistive and inductive component of the textile inductor. The textile inductor has a self-resonance frequency (indicated by a dot in the figures) at around 58 MHz.

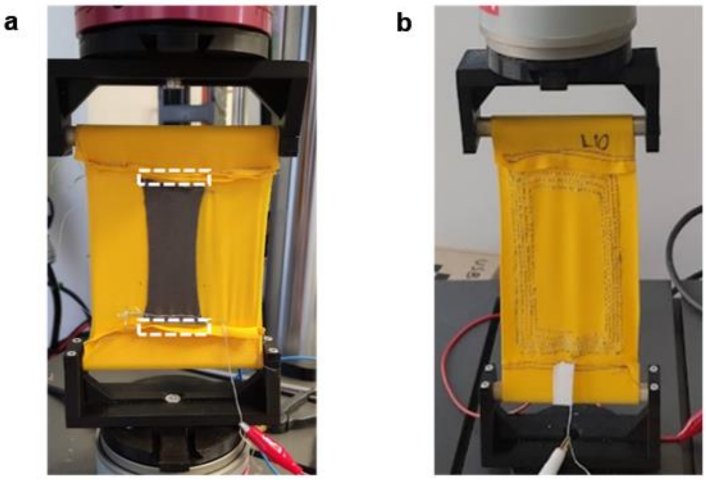

Figure S3. Setup for the mechanical characterization tests. a) Textile capacitor mounted on the UTM with the help of sewn loops anchored to the 3D printed fixtures. The textile capacitor electrodes (grey conductive Spandex) between these sewn loops (the region between the white dashed areas) underwent considerable strain at higher strain levels. b) Textile inductor mounted on the UTM with the help of sewn loops anchored to the 3D printed fixtures.

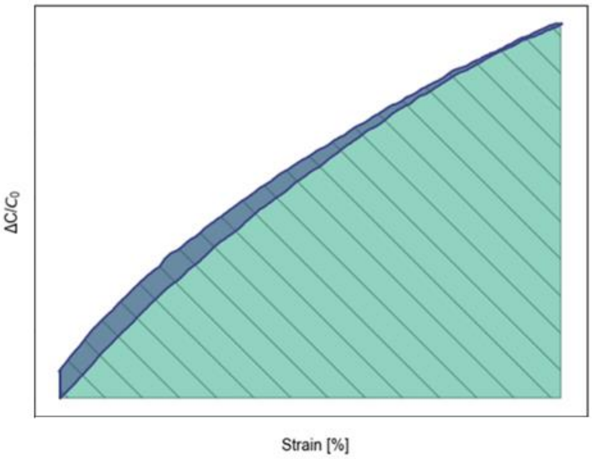

Figure S4. Calculation of hysteresis. A representative figure indicating calculation of hysteresis as the ratio between the area enclosed by the extension-relaxation loop (blue) normalized by the area under the curve of the extension curve (hatched green).

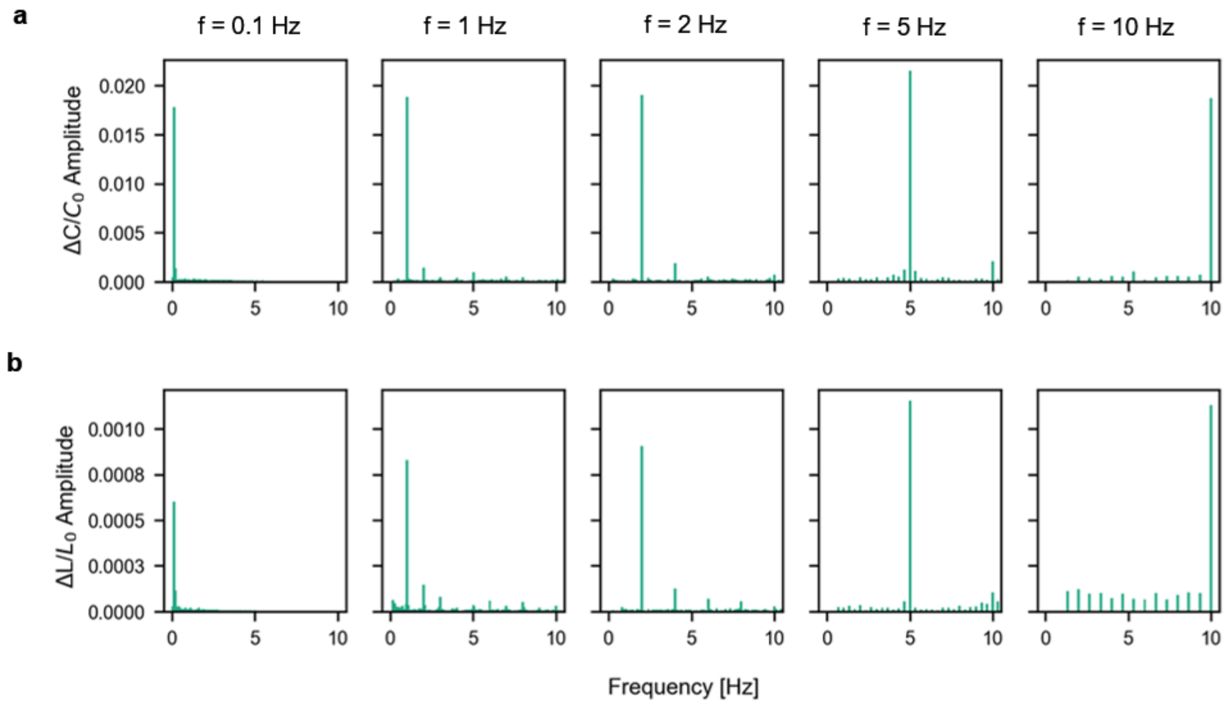

Figure S5. Response of textile elements to different strain rates in the frequency domain. Fast Fourier Transform (FFT) analysis of the electrical response to a fixed-amplitude sinusoidal strain waves of different frequencies for the a) textile capacitor, b) textile inductor.

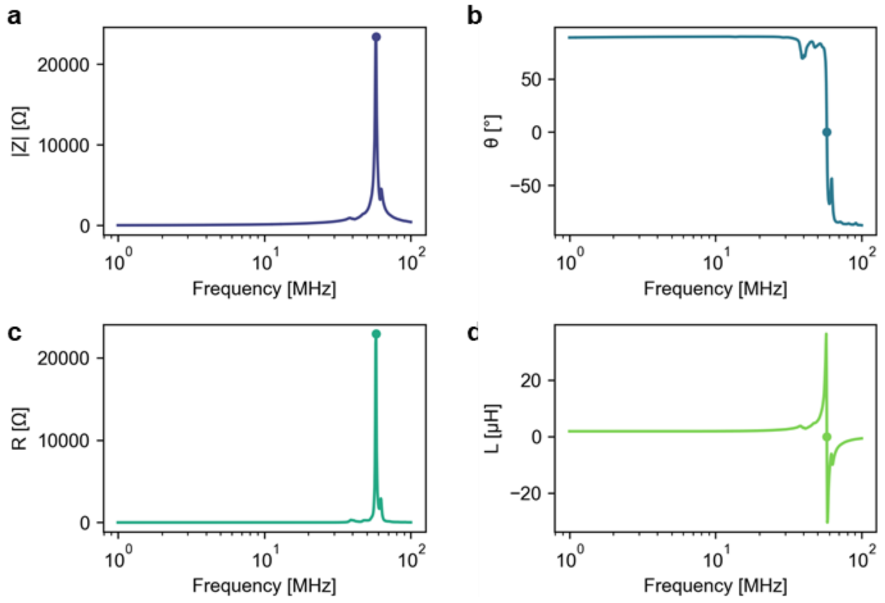

Figure S6. Impedance characterization of the external reader inductor used with the VNA. a,b) Magnitude and phase of external reader inductor's impedance. c,d) Magnitude of the resistive and inductive component of the external reader inductor. The inductor has a self-resonance frequency (indicated by a dot in the figures) at around 57 MHz.

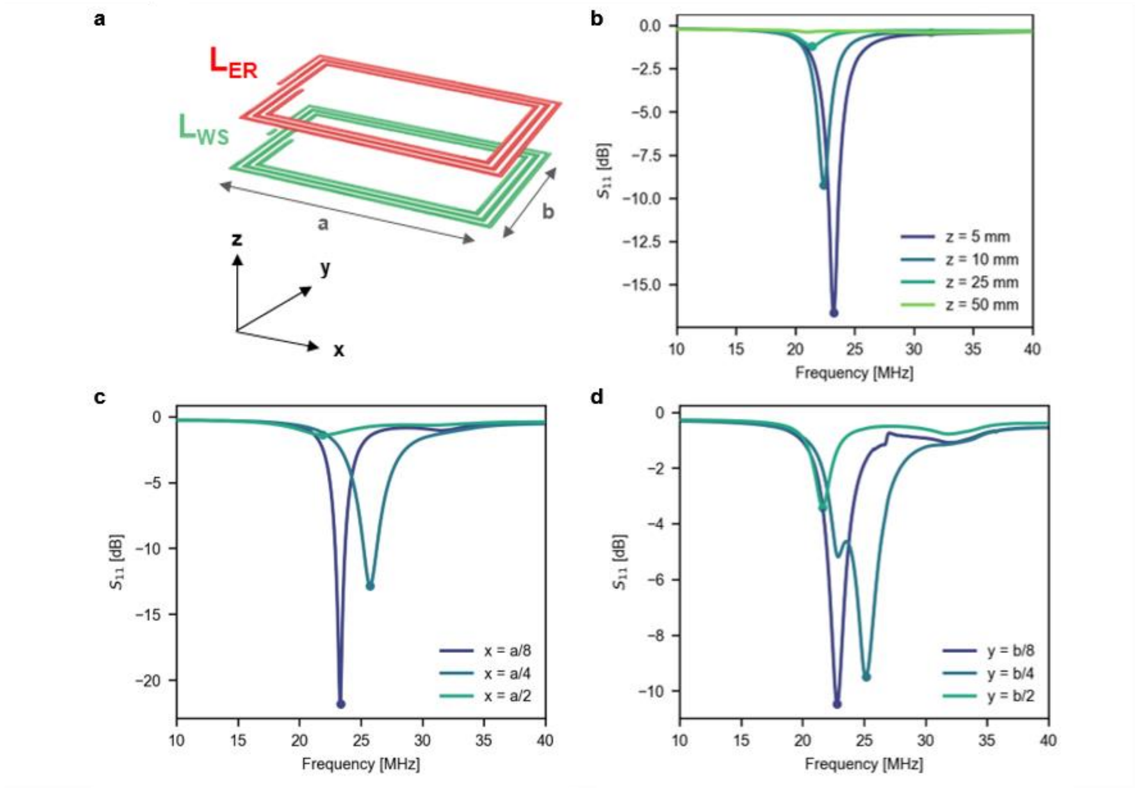

Figure S7. Influence of the reciprocal position of the textile inductor and the external reader inductor on wireless reading. a) Schematic depicting the relative position between inductors. b, c, d) Variation in S_{11} profile due to the misalignment between inductors in z, x and y directions.

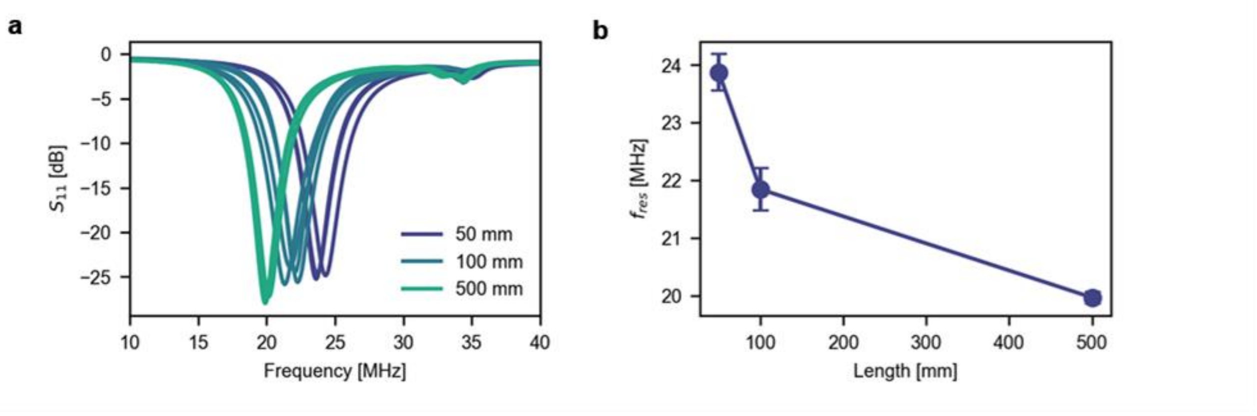

Figure S8. Influence of transmission line length. a) S_{11} profiles of the readings on the wearable sensor with transmission line of three different lengths between the textile capacitor and inductor. b) Representation of f_{res} with respect to the transmission line length.

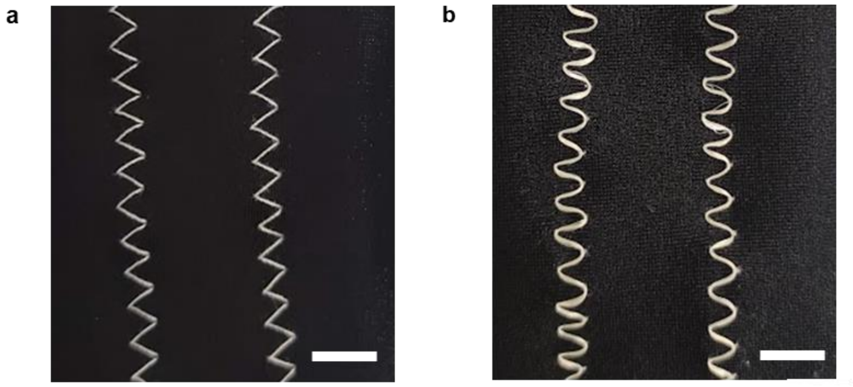

Figure S9. Transmission line. a) Front (polyester thread) and b) back (Liberator thread) close-up pictures of the loose zig-zag stitched transmission line (scale bar 1 cm).

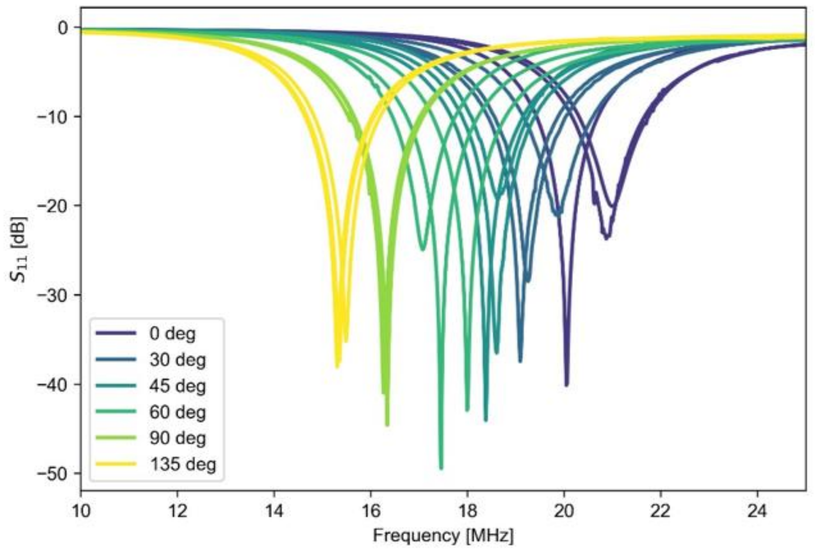

Figure S10. Knee bending test. Profiles of the S_{11} scattering parameter for 6 different angles (3 measurements each).

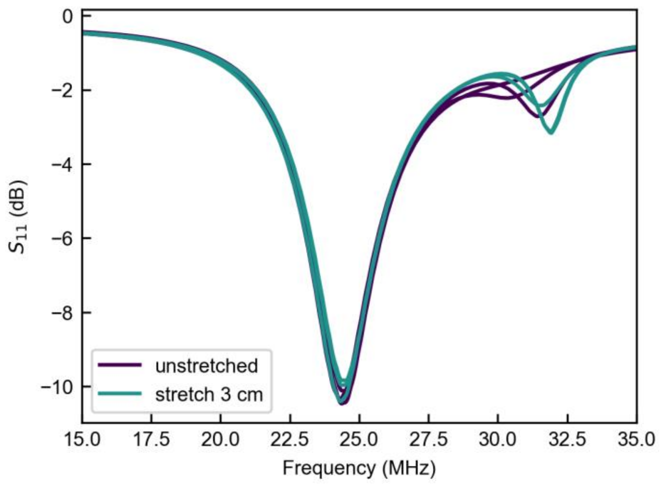

Figure S11. Influence of transmission line on resonance frequency of the sensing system. Profile of S_{11} scattering parameters obtained by stretching the cloth anchoring the transmission line to 3 (the graph shows three separate measurements).

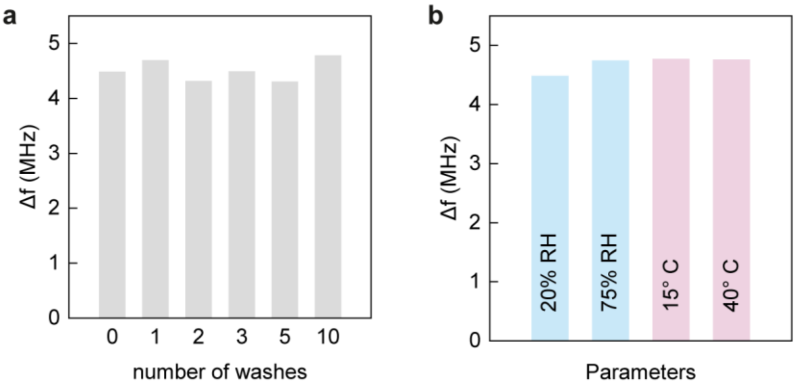

Figure S12. Sensor performance after washing and under different environmental conditions. Variation in sensor response (Δf) registered for a 30% strain using VNA as a reader a) after repeated washing of the garment, b) at different humidity and temperature.

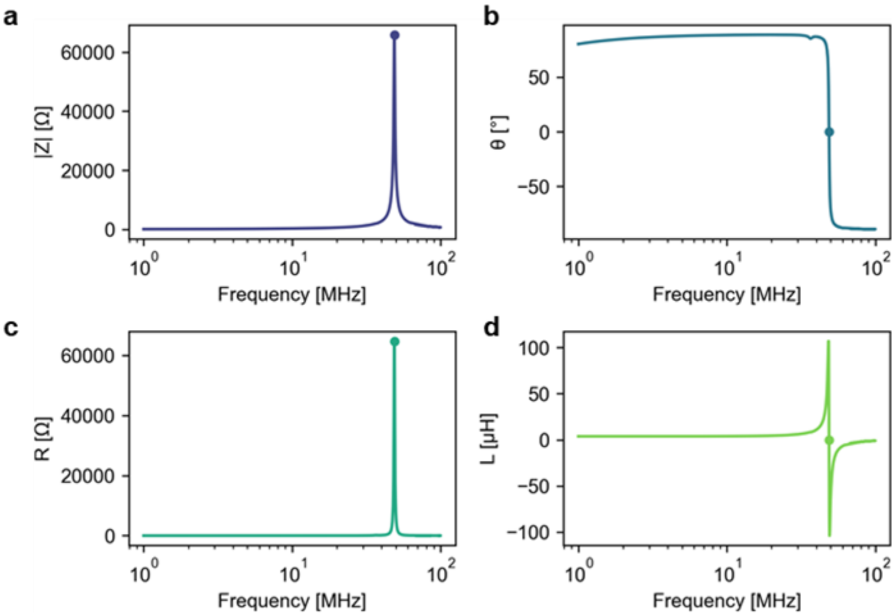

Figure S13. Impedance characterization of the textile inductor used with the fReader. a,b) Magnitude and phase of the textile inductor's impedance. c,d) Magnitude of the resistive and inductive component of the textile inductor. The inductor has a self-resonance frequency (indicated by a dot in the figures) at around 40 MHz.

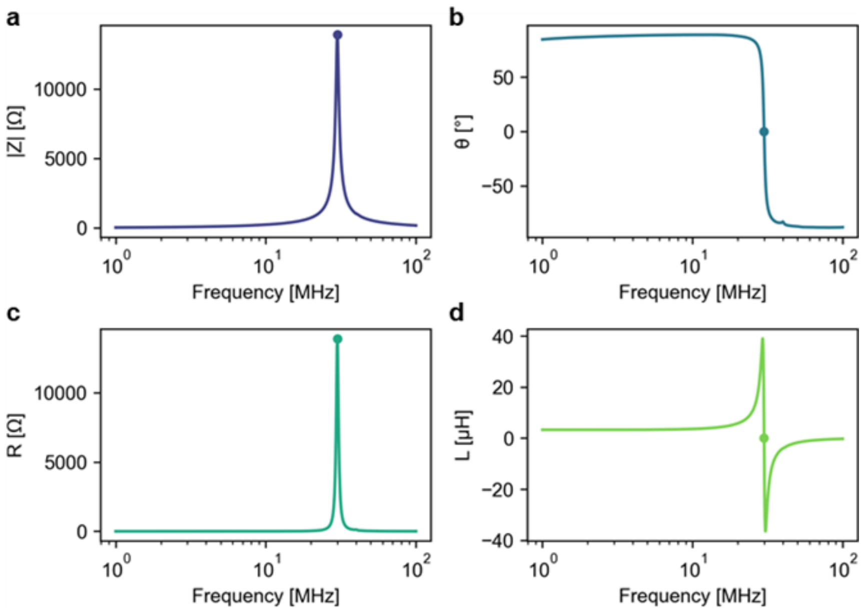

Figure S14. Impedance characterization of the commercial inductor of the fReader. a,b) Magnitude and phase of the fReader inductor's impedance. c,d) Magnitude of the resistive and inductive component of the fReader inductor. The inductor has a self-resonance frequency (indicated by a dot in the figures) at around 30 MHz.

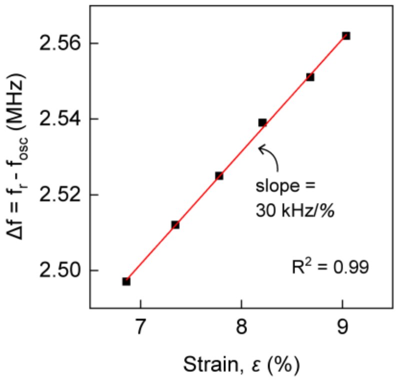

Figure S15. Frequency changes with the textile capacitor under strain. Changes in frequency registered by the fReader with textile capacitor under different strains. f_r and f_{osc} correspond to the frequency obtained with no textile capacitor, and with the textile capacitor under a particular strain.

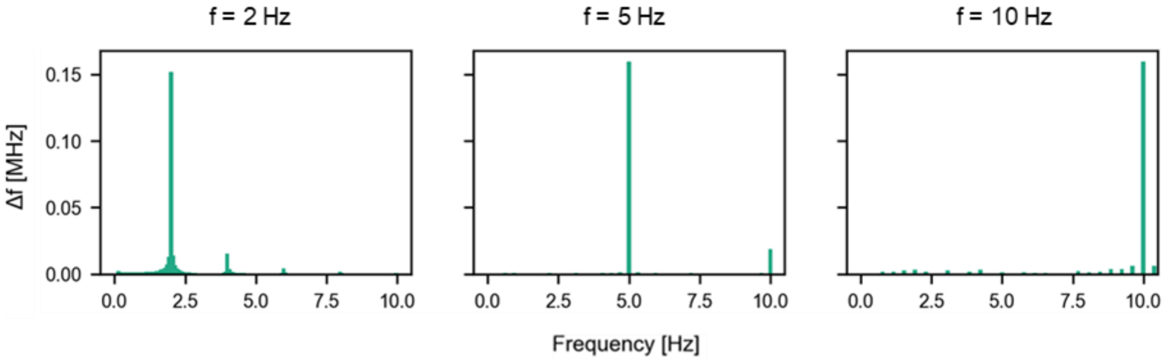

Figure S16. fReader's response with textile capacitor subjected to different strain rates. Fast Fourier Transform (FFT) analysis of the fReader's response to a fixed-amplitude sinusoidal strain waves of different frequencies imposed on the textile capacitor.

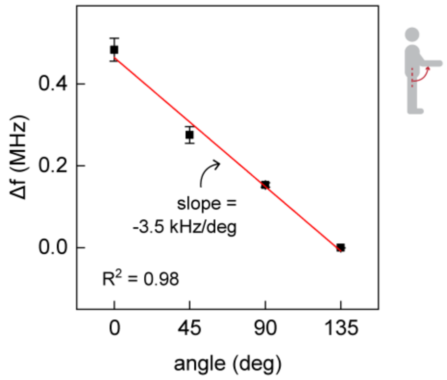

Figure S17. Frequency changes with the LC textile sensor for different degrees of arm movement. Variation in the frequencies registered by the fReader with the LC textile sensor on the sport shirt (above the elbow) for different degrees of arm movement. The data is represented as an average of three independent readings with standard deviation.
